# Notes and News

**Published:** 1906-05-12

**Authors:** 


### Notes and News.

A new wing for children is about to be added to the Hunstanton Convalescent Home. The cost is estimated at £5,000.

The London Homœopathic Hospital, Great Ormond Street, W.C., has received a legacy of £250, less legacy duty, from the late Henry Hargreaves Bolton, Esq.

A sanatorium for consumption has been erected at Barrasford in Northumberland, but is not yet occupied owing to lack of funds. The buildings are arranged in a semicircle, so that shelter and sunlight are best secured, and ample provision for air has been provided.

A new infectious diseases hospital at Fazakerley, for Liverpool, has been opened. The site is 118 acres in extent, is five miles from the centre of the city, and cost £39,915. The buildings are 24 in number, and cover about 35 acres. The cost was about £121,475. There are nine pavilions and four isolation blocks, with a total accommodation for 350 patients.

